# Comment on ‘Development of virtual ophthalmic surgical skills training’

**DOI:** 10.1038/s41433-022-02053-y

**Published:** 2022-04-06

**Authors:** Charles O’Donovan

**Affiliations:** Royal Eye Unit, Kingston Hospital NHS Foundation Trust, London, UK

**Keywords:** Education, Outcomes research

Thank you to authors of the article ‘Development of virtual ophthalmic surgical skills training’ for an interesting perspective on how ophthalmic surgical skills can be taught successfully online to a diverse student cohort [1]. The authors conclude that virtual delivery of ophthalmic surgical skills can widen accessibility and participation to students.

As a second year Ophthalmology Speciality Trainee, I can appreciate the immense adverse impact COVID-19 has had, particularly on surgical training where the majority of elective surgery was postponed during the initial acute phase of the pandemic. A recent Royal College of Ophthalmologists (RCOphth) trainee survey found over half (55%) lacked surgical opportunities to achieve objectives of their placement [2].

Simulation has already proved extremely effective in ophthalmology training with evidence showing that trainees who have spent time using the EyeSi have reduced complication rates and a shorter learning curve when they transfer to live cataract surgery [3].

Given access to EyeSi simulators is variable across different deaneries, virtual surgical skills training provides an ideal cost-effective, safe solution for developing surgical skills amongst trainees. Virtual training can speed up the learning curve through improving manual dexterity, the basics of instrument handling and suturing skills.

Limited information is provided in the article on the training received prior to the course for the trainers, this is essential for success. Furthermore, it is crucial trainers and consultants have dedicated time and renumeration to support these virtual simulation sessions.

This method of training also requires adequate digital literacy for both trainees and trainers. This is defined as the ability to use digital technology, communication tools or networks to locate, evaluate, use and create information [4]. Trainers need to become fluent with the digital platform to maximise the pedological experience for trainees [5]. Additionally, this method of teaching requires technological prerequisites, particularly stable WiFi connection and adequate camera angles.

To conclude virtual ophthalmic surgical skills is an efficient and convenient way of learning. It can be harnessed in ophthalmology training as an additional (not alternative) method of teaching in overcoming limited surgical opportunities due to COVID-19 and assist trainees in becoming more confident surgeons.
